# Advancing Pharmacy Practice through an Innovative Ambulatory Care Transitions Program at an Academic Medical Center

**DOI:** 10.3390/pharmacy8010040

**Published:** 2020-03-12

**Authors:** Jamie Cavanaugh, Nicole Pinelli, Stephen Eckel, Mark Gwynne, Rowell Daniels, Emily M. Hawes

**Affiliations:** 1 Department of Medicine, University of North Carolina (UNC) School of Medicine, Chapel Hill, NC 27599, USA; jamie_cavanaugh@med.unc.edu; 2 UNC Eshelman School of PharmacyChapel Hill, NC 27599, USA; nickipinelli@unc.edu (N.P.); sfeckel@email.unc.edu (S.E.); rowell.daniels@unchealth.unc.edu (R.D.); 3 Department of Pharmacy, UNC Health, Chapel Hill, NC 27514, USA; 4 UNC Health Alliance, UNC Health, Morrisville, NC 27560, USA; Mark.Gwynne@unchealth.unc.edu; 5 Department of Family Medicine, UNC School of Medicine, Chapel Hill, NC 27599, USA

**Keywords:** care transitions, pharmacist, medication reconciliation, hospital follow-up, ambulatory care, primary care

## Abstract

Hospital readmissions are common and often preventable, leading to unnecessary burden on patients, families, and the health care system. The purpose of this descriptive communication is to share the impact of an interdisciplinary, outpatient clinic-based care transition intervention on clinical, organizational, and financial outcomes. Compared to usual care, the care transition intervention decreased the median time to Internal Medicine Clinic (IMC) or any clinic follow-up visit by 5 and 4 days, respectively. By including a pharmacist in the hospital follow-up visit, the program significantly reduced all-cause 30-day hospital readmission rates (9% versus 26% in usual care) and the composite endpoint of 30-day health care utilization, which is defined as readmission and emergency department (ED) rates (19% versus 44% usual care). Over the course of one year, this program can prevent 102 30-day hospital readmissions with an estimated cost reduction of $1,113,000 per year. The pharmacist at the IMC collaborated with the Family Medicine Clinic (FMC) pharmacist to standardize practices. In the FMC, the hospital readmission rate was 6.5% for patients seen by a clinic-based pharmacist within 30 days of discharge compared to 20% for those not seen by a pharmacist. This transitions intervention demonstrated a consistent and recognizable contribution from pharmacists providing direct patient care and practicing in the ambulatory care primary care settings that has been replicated across clinics at our academic medical center.

## 1. Introduction

Health care reform is placing an emphasis on improving the quality of care delivered to our patients. New pharmacy practice models, demonstrating enhanced quality of care through replicable, scalable, and sustainable methods, are needed for health systems and hospitals across the United States to meet these challenges. One suggested area for improvement is reducing preventable hospital readmissions. The following describes the development of a systematic process and the subsequent dissemination of an innovative ambulatory pharmacy practice model that has significantly reduced readmissions and saved over $1 million in avoidable costs.

Hospital readmissions are common, with an estimated 18.8% of Medicare patients readmitted to the hospital within 30 days [1]. Many of these hospital readmissions are preventable, leading to unnecessary burden on patients, families, and the health care system [2]. In addition, the Affordable Care Act implemented by the Centers for Medicare and Medicaid Services (CMS) financially penalizes individual institutions with 30-day readmission rates higher than their peers. Hospitals with elevated readmission rates are at risk for financial penalties from CMS [3].

Although programs evaluating pharmacist-led medication reconciliation have demonstrated reduced readmissions, there is significant heterogeneity and most are inpatient or telephone interventions [4,5,6,7,8,9]. Limited data, outside of our institution, exist showing the impact of pharmacy-coordinated, interdisciplinary, outpatient clinic-based care transition programs [10,11,12,13,14]. Consequently, there is a critical need for health care institutions to identify outpatient pharmacy best practices for improving the care transition of patients from the inpatient to ambulatory care environment. In addition, this supports many of the key findings from the American Health-System Pharmacists (ASHP) Foundation Ambulatory Conference and Summit Consensus Recommendations [15]. These included ‘establishing consistent and sustainable models for seamless transitions across the continuum of care’ and ‘demonstrating measurable and meaningful impact on individual patient and population outcomes’.

Pharmacists are the ideal medical professional to reduce readmission rates and limit financial consequences for institutions because two-thirds of hospital readmissions are secondary to medication adverse events [16]. The pharmacy profession has placed a major emphasis on expanding the role of the pharmacist in the ambulatory care setting. However, to fully realize the impact of these professionals, their activities need to be rigorously evaluated and published.

In 2011, 483 Medicaid patients discharged from a variety of hospitals received care through the Internal Medicine Clinic (IMC) at the University of North Carolina (UNC) in Chapel Hill, North Carolina; of these, 91 (18.8%) were readmitted within 30 days. Since the 30-day readmission rate in our Medicaid population was one of the highest across our state, we hypothesized that there is a significant opportunity to use pharmacists to improve care transition issues among IMC patients. To that end, our project’s purpose was to (1) develop an interdisciplinary, outpatient clinic-based care transition intervention aimed at reducing 30-day hospital readmission rates; (2) examine the impact of this intervention on clinical, organizational, and financial outcomes; and (3) describe the role of the pharmacist and the significance of this program to pharmacy practice advancement.

## 2. Significance of the Ambulatory Care Transitions Program to our Health System

### 2.1. Environmental Scan

An interdisciplinary team completed a chart review and an analysis of the baseline environment during development of the care transition program in order to identify opportunities for improvement in managing patients at increased risk for hospital readmission. At baseline, approximately 270 IMC patients were admitted to our health care system each month, and 20% were readmitted within 30 days. Chart review and process mapping identified several key opportunities for improvement including: (1) standardization of clinic visit content to include pharmacotherapy as a primary component of the care transition intervention; (2) involvement of care management; and (3) timely follow-up after hospital discharge. The high readmission rate demonstrated for IMC patients, coupled with the financial implications to our institution, served as the justification for providing the critical support needed to further develop an intervention aimed at reducing 30-day hospital readmission rates. A chart review of hospital readmissions revealed medication errors and medication-related adverse events as contributing factors leading to the identification of the pharmacist as a critical care team member. Furthermore, pharmacies were asked to serve as the leader in this interdisciplinary collaboration to develop and implement this intervention.

### 2.2. Program Development

During program development, the IMC established a readmission leadership team that included pharmacists, physicians, care managers, support staff, clinic management, and hospital quality improvement leadership. The readmission leadership team adopted the Institute for Healthcare Improvement’s State Action on Avoidable Rehospitalizations (IHI STAAR) guide as the framework for the intervention [6]. Pharmacies were directly involved in developing the ideal process map and a detailed plan for standardizing the pharmacy-coordinated, interdisciplinary, outpatient clinic-based care transitions program, which was adapted from the suggested clinic visit components included in the IHI STAAR guide [16].

The standardized care transition intervention schedules made IMC patients eligible for a 60-minute hospital follow-up appointment within 14 days of discharge [11]. Established IMC patients regardless of the reason for admission or payer status were included. Patients admitted for scheduled chemotherapy, outpatient procedures (i.e., colonoscopy), and obstetrics were excluded. We also excluded patients discharged to hospice. If patients were discharged to a rehabilitation or skilled nursing facility, we aimed to see the patient within 14 days of discharge from that facility rather than hospital discharge. The clinical pharmacist coordinated the hospital follow-up visit in the IMC. Key pharmacy features of the intervention included: (1) medication management through a collaborative practice agreement; (2) identification of medication-related problems through performing medication reconciliation; (3) medication education using teach-back methodology; (4) identification of patient self-reported reasons for hospital admission; (5) identification of any barriers to care; (6) assistance with referral to support services as applicable; and (7) documentation to ensure continuity of care. The study was approved by the University of North Carolina (UNC) Institutional Review Board (study number 15-2812 and 13-3867).

### 2.3. Program Implementation

The program was implemented in two phases: pilot and full capacity. The pilot phase began in March 2012, following clinic leadership approval. During the pilot phase, the IMC dedicated 6–10 hospital follow-up appointments per week to the care transition intervention. Goals of the pilot phase were to (1) refine the clinic process and intervention content and (2) develop a standardized documentation template through feedback gathered from IMC providers and patients. Following completion of the pilot phase, our health system leadership, in conjunction with a community partner, provided pharmacist support in order to demonstrate the impact of this program on clinical, organizational, and financial outcomes. Consequently, the hospital follow-up clinic was expanded to full capacity in September 2012. During the full capacity phase, the IMC clinic dedicated 27 hospital follow-up appointments per week to the care transition intervention; of these, 21 (78%) were coordinated by the dedicated clinical pharmacist. In order to contribute to medical education, the remaining six hospital follow-up appointments were coordinated by medical residents who received training by the clinical pharmacist.

### 2.4. Coordination with Other Disciplines

During program implementation, the clinical pharmacist worked closely with the inpatient quality improvement team to obtain a daily discharge list of IMC established patients. This integration was critical to our success as it provided an alert to IMC care management of hospitalizations and ensured that the dedicated appointments were utilized appropriately. IMC care management was responsible for contacting each patient to ensure an appointment was scheduled within 14 days of discharge and address any immediate barriers to care, such as difficulty affording discharge medications or transportation to follow-up visits. The coordination with inpatient care managers increased inpatient provider awareness of the new IMC hospital follow-up program and greatly improved clinic visit scheduling prior to hospital discharge. Finally, the education of IMC attending and resident physicians, as well as nursing staff, ensured consistent clinic processes and intervention delivery.

### 2.5. Continuous Quality Improvement

The IMC readmission leadership team met at least monthly to review progress toward both clinic-level and health care system-level goals. Control charts were developed for monthly readmission rates, time to follow-up, follow-up provider type (as a marker of dedicated hospital follow-up appointment type utilization), and no-show rates. Each chart was carefully reviewed by the leadership team. Goals were established and agreed upon at the clinic and health care system levels. Then, the model for improvement and small tests of change were utilized for continuous process refinement [17].

## 3. Demonstration of Improvements

The IMC hospital follow-up program, coordinated by an ambulatory care clinical pharmacist, has been in place since March 2012. It has been exceptionally well received by health system leadership, inpatient and outpatient medical staff, care management, and the pharmacy department. The research group evaluated outcomes in patients receiving the pharmacist-coordinated transitions intervention to those who did not (usual care). The statistical analysis completed has been fully described previously [11].

This evaluation revealed that an ambulatory pharmacist managing a post-acute follow-up program can lead to improved clinical, organizational, and financial outcomes, including:

- Standardization of the care transition intervention
- Identification of medication-related problems
- Decreased time to hospital follow-up
- Reduction in readmission rates and emergency department (ED) rates
- Estimated cost savings through avoided readmissions
- Improved reimbursement through appropriate billing of the transitions of care codes

### 3.1. Standardization of Visit Content

**Figure 1:**
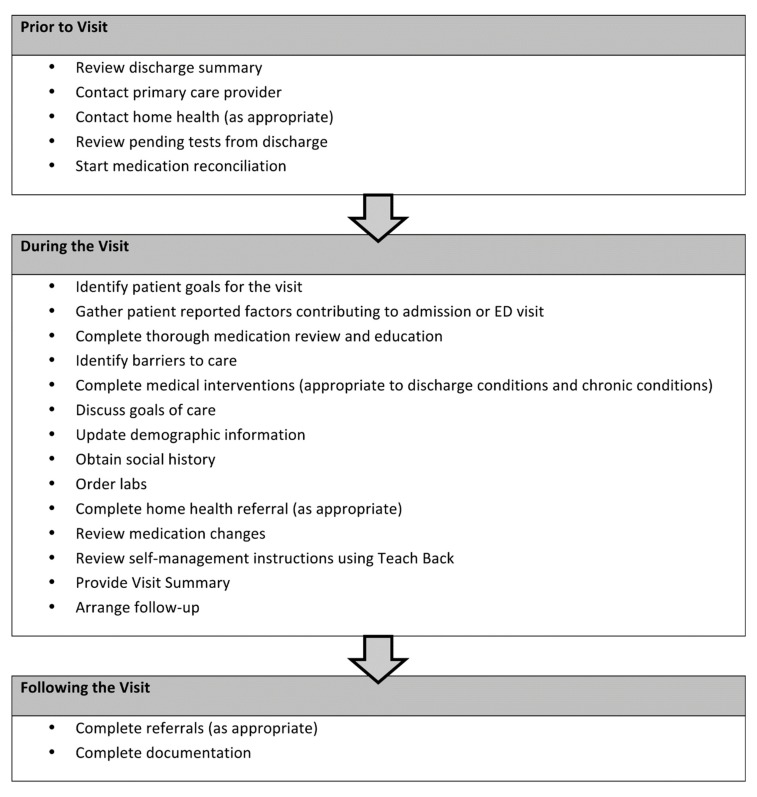
Key visit components.

The clinical pharmacist was able to standardize the care transition intervention based on an adaptation of the IHI STAAR guide checklist [16]. Figure 1 depicts the key visit components that are delivered in the presence or absence of the clinical pharmacist. The significance of this outcome to our health system is that we have identified the core elements of a hospital follow-up program that are associated with improved organizational and financial outcomes. These core elements serve as the basis for broad-scale deployment in the provision of clinical patient care services and can ensure consistency in delivery of the intervention across patients at our institution.

### 3.2. Identification of Medication-Related Problems

Table 1 depicts the common interventions of the clinical pharmacist during the direct patient care visit. The most common activity is identification of medication non-adherence, followed by the addition or discontinuation of medications, disease state monitoring, and smoking cessation counseling.

### 3.3. Decreased Time to Hospital Follow-up

Compared to usual care, the program was associated with a 5 and 4-day decrease in the median time to IMC or any clinic follow-up, respectively (Table 2) [11]. The program was also associated with an increased proportion of discharged patients attending a follow-up visit within 30 days. We attribute these findings to the increased availability of appointments coupled with the role of care management in the scheduling of appointments, addressing transportation barriers, and conducting reminder calls.

### 3.4. Reduction of Readmission Rates and ED Visits

The program was associated with reduced health care resource utilization. Compared to usual care, by including a pharmacist in the hospital follow-up visit, the program significantly reduced all cause hospital readmission rates and the composite endpoint of health care utilization (readmission and ED rates) at 30 days (Table 2) [11]. Interestingly, the benefits of this program on health care utilization were sustained at 90 days.

### 3.5. Estimated Cost-Savings through Avoided Readmissions

Based upon our survival analysis, one 30-day hospital readmission was prevented for every seven patients seen in the hospital follow-up clinic. Over the course of one year, this program can prevent 102 30-day hospital readmissions with an estimated cost reduction of $1,113,000 per year [18]. In addition, it can reduce the institution’s overall rate of readmissions and minimize the chance for receiving the financial penalties for poor-performing hospitals.

### 3.6. Improved Reimbursement through Appropriate Billing of the Transitions of Care Codes

Pharmacist involvement in the hospital follow-up program has allowed for improved billing for services through the Medicare Transitional Care Management (TCM) reimbursement codes. While the claim must be submitted under a Medicare recognized provider, these codes provide higher Relative Value Units (RVUs) and subsequently higher reimbursement rates to reflect the involvement of multiple providers. Therefore, pharmacist-provided patient care in the IMC allowed for an enhanced billing for transition services.

## 4. Significance of the Program to Pharmacy Practice Advancement

For nearly 25 years, delivery system re-engineering in hospital and health system pharmacy practice has been primarily based on the incremental addition of new clinical patient care services or programs [19]. However, broader and more systematic changes have not occurred. This may be, in part, because we still have not identified a consistent approach to pharmacy practice. Addressing this challenge will be critical if we are to achieve the magnitude of change being called for in hospital and health system pharmacy practice by the Practice Advancement Initiative (PAI, formerly Pharmacy Practice Model Initiative) and the recently concluded Ambulatory Care Summit [20]. Thus, there is a clear mandate for change in hospital and health system pharmacy practice to develop a consistent model of practice that can be applied across various practice settings and clinical specialty areas. The current program described in this project has demonstrated improvement of clinical, organizational, and financial outcomes; however, the greatest impact of this program has been in the development of core elements of a pharmacy-coordinated, interdisciplinary, outpatient clinic-based care transition intervention for broad-scale deployment across practice settings and specialty areas. Indeed, the core elements of our program have been extended beyond the IMC and adapted across several practice settings and specialty areas within our institution.

### 4.1. Coordination with Additional Outpatient Clinics

Our Family Medicine Clinic (FMC) has coordinated with the IMC in developing and adapting their own transitions of care intervention. Similar to the IMC program development process, the FMC first established the efficacy of pharmacy involvement in transitional care. In the FMC, this was established through a prospective, randomized, open-label, pilot study with the objective of evaluating the effect of a pharmacy clinic visit focused on medication reconciliation and patient education after hospital discharge. Of the 61 subjects included in the study, 33 (54%) had medication discrepancies identified at discharge. More medication discrepancies were resolved in subjects randomized to the pharmacist intervention arm compared with the usual care of seeing a medical provider following discharge (50% versus 9.5%, *p* = 0.015). Patients randomized to the intervention had significantly fewer 30-day re-hospitalization and ED visits compared with the usual care arm (0% versus 40.5%, *p* < 0.001) [14]. Due to the success of this project and the focus on reducing hospital readmission rates, the FMC began piloting an outpatient interdisciplinary team-based transitions process of care in September 2011. The process included a visit with a pharmacist immediately followed by a primary care provider (PCP) visit and a care manager. Linking the pharmacist and medical provider appointment decreased the pharmacy clinic no-show rates from 58% to 25%. During this pilot phase, the transitional care visits were limited to a subset of established adult FMC patients discharged specifically from our institution’s Family Medicine Inpatient Service (FMIS). The pilot included 36 patients (an estimated 12.5% of potentially eligible patients). The transitions process of care visits reduced 30-day readmission rates from 27.3% to 16.7%. The pharmacist identified an average of 3.6 medication-related problems per patient.

Throughout the FMC pilot period, the pharmacist led weekly interdisciplinary quality improvement meetings to conduct sequential Plan, Do, Study, Act (PDSA) cycles in order to improve the process. For example, after modifying the appointment reminder phone call to include a detailed request for patients to bring medication-related items, 70% of patients brought medication bottles to the appointment.

Further, the pharmacists at IMC and FMC collaborated to standardize the pharmacy intervention between practices. This included standardized medication reconciliation, coordinated quality improvement efforts, shared resources, and streamlined processes.

In September 2013, the FMC expanded the transitions process of care intervention to all patients discharged from the FMIS at moderate to high-risk for readmission based upon institutional definition (Table 3). Unfortunately, difficulty coordinating the clinic availability of the PCP and pharmacist for the joint appointment led to fewer than anticipated patients receiving the full intervention. As part of the quality improvement process, the impact of process-specific factors on 30-day readmission rates were analyzed in 107 patients at moderate to high risk for readmission (Table 4 and Table 5). The lower 30-day readmission rates in patients seen by an outpatient pharmacist at the FMC after discharge (Table 6) and the aforementioned success of the IMC program led clinic leadership to prioritize pharmacist follow-up starting in June 2014 [12]. Modeling after the IMC, patients of the FMC who are at moderate to high risk for readmissions are now scheduled with a pharmacist and any medical provider (not necessarily the patient’s specific PCP) within 7 days of discharge.

### 4.2. Coordination with Health Care System Initiatives

**Figure 2:**
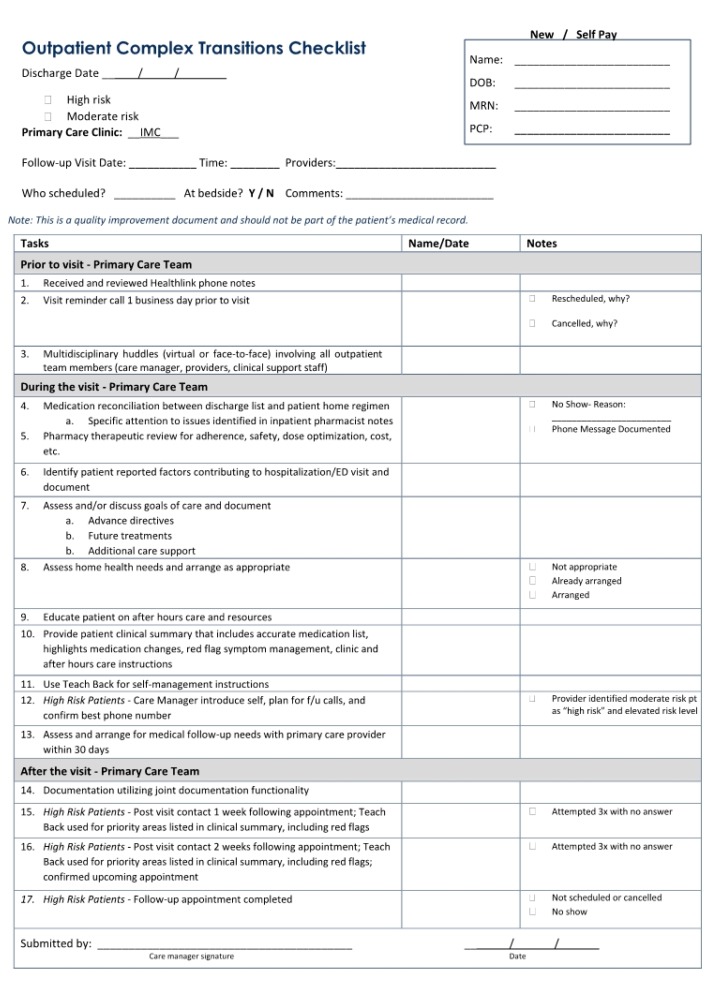
Outpatient checklist.

In September 2013, our health care system coordinated a systematic broad-scale quality improvement initiative to align all care transitions interventions at our institution. This quality improvement initiative resulted in the development of inpatient and outpatient interdisciplinary processes of care checklists for patients at high or moderate risk for readmission based upon institutional definitions. For the outpatient checklist (Figure 2), clinical pharmacists from the IMC and FMC were asked to serve as care transition champions along with physicians, care managers, and nurses in the development of the process of care checklist. Core elements of the interdisciplinary outpatient clinic-based care transitions program have been endorsed across the entire institution as a standard of care, and the process implementation has begun in outpatient specialty practices, including cardiology and geriatrics. These were locations that already had pharmacists in place who are now also involved in transitions of care visits. Additionally, the IMC and FMC clinical pharmacists have contributed to the development of standardized processes of care for the inpatient checklist, including provisions for: (1) handoffs between inpatient and outpatient pharmacists, physicians, and care managers; (2) hospital follow-up clinic appointment scheduling prior to hospital discharge; and (3) the coordination of post-discharge phone calls to patients to avoid duplication of services.

We have identified and documented features and significant outcomes of a pharmacy practice model that can be adapted across a variety of ambulatory care settings, which is likely to contribute to pharmacy practice advancement. A key component of our system’s implementation has been through shifting our focus from the incremental addition of clinical patient care services to a broader system redesign. In alignment with the American Society of Health-System Pharmacists Practice Advancement Initiative (PAI), we have (1) described the practice model (PAI objective 1); (2) determined core elements of a pharmacy-coordinated, interdisciplinary, outpatient clinic-based care transitions program (PAI objective 2); and (3) provided valuable insights into strategies to support implementation in diverse settings (PAI objectives 3–5) [20].

## 5. Conclusions

In today’s health care arena, all services must justify how they bring value to the organization. In addition, demonstrating that a single activity can permeate an institution’s culture such that it is utilized in various areas is critical. This report describes the impact of a pharmacy-coordinated, interdisciplinary outpatient clinic-based care transition intervention that is associated with improved clinical, organizational, and financial outcomes, including reduced 30-day hospital readmission rates. We have also demonstrated a consistent, recognizable contribution from pharmacists providing direct patient care and practicing in the ambulatory care setting at our institution. This process of care is a model that has been shown to be replicable across other hospital clinics. We believe it to be a model that can also be adopted by other health systems and hospitals to utilize ambulatory-based pharmacists in an effort to significantly reduce readmissions.

Not only does this practice model mirror the profession’s goal of advocacy and the expanding role of the pharmacist in patient care in the ambulatory care setting, but it also aids an organization’s approach to meeting quality goals in the new health care arena.

## Figures and Tables

**Table 1:** Interventions during Internal Medicine Clinic (IMC) hospital follow-up visits.

Intervention	n (%) *
Identification of cost issues	28 (28.0)
Identification of non-adherence	51 (51.0)
Medication adjustments	
Therapeutic switch	4 (4.0)
Medication addition	61 (39.0)
Medication discontinuation	30 (27.0)
Dose increase	17 (17.0)
Dose decrease	19 (18.0)
**Referrals**	
Specialist	16 (14.0)
Home health	3 (3.0)
Social worker	12 (12.0)
Emergency department	3 (3.0)
Behavioral counseling	2 (2.0)
Financial counseling	2 (2.0)
Follow up with outpatient pharmacist	2 (2.0)
**Laboratory Monitoring**	
Medication monitoring	16 (16.0)
Disease state monitoring	45 (33.0)
**Vaccinations (influenza, Tdap, PPSV)**	12 (10.0)
**Lifestyle counseling**	
Smoking cessation	30 (30.0)
Alcohol cessation	5 (5.0)
**Interventions per visit (median, IQR)**	3 (2–5)

* Values reported indicate the total number of interventions and the percent of visits in which the intervention occurred.

**Table 2:** IMC readmissions, emergency department (ED) visits, composite outcomes and time to follow-up. * *p* < 0.05.

	Intervention (%) (n = 54)	Usual Care (%) (n = 54)	*p*-Value
30-day readmissions	5 (9)	14 (26)	0.023*
90-day readmissions	10 (19)	24 (44)	0.004*
30-day ED visits	6 (11)	12 (22)	0.121
90-day ED visits	11 (20)	17 (31)	0.188
30-day composite (ED or readmission)	10 (19)	24 (44)	0.004*
90-day composite (ED or readmission)	18 (33)	32 (59)	0.007*
Days to first IMC follow-up (Median)	7 (IQR 6, 11)	12 (IQR 7.5, 25.5)	<0.001*
Days to first clinic follow-up (Median)	6.5 (IQR 5, 10)	10.5 (IQR 7, 17)	<0.001*
Hospital follow-up within 30 days	54 (100)	46 (85)	0.003*

**Table 3:** Health system hospital readmission risk classification.

Moderate Risk	High Risk
Two or more hospital admissions in the last year OR at least two chronic conditions * with no regard to medications	Three or more hospital admissions in the last year OR at least three chronic conditions * AND at least 10 medications

* Chronic conditions as stated above include: heart failure, chronic obstructive pulmonary disease, pneumonia, dementia, depression, diabetes, chronic kidney disease, prior myocardial infarction.

**Table 4:** Family Medicine Clinic (FMC) baseline demographics.

Characteristic	Number of Patients (%) (n = 107)
Mean age [years ± SD]	54.9 ± 16.9
Gender-Female	58 (54.2)
Race	
Caucasian	58 (54.2)
African-American	41 (38.3)
Other	8 (7.5)
Ethnicity	
Non-Hispanic	101 (94.4)
Primary language	
English	103 (96.3)
Comorbidities	
Depression	55 (51.4)
Diabetes	46 (43.0)
CHF	30 (28.0)
CKD	27 (25.2)
COPD	25 (23.4)
AMI	10 (9.3)
PNA	7 (6.5)
Dementia	2 (1.9)

Notes: SD = standard deviation; CHF = Congestive Heart Failure; CKD = Chronic Kidney Disease; COPD = Chronic Obstructive Pulmonary Disease; AMI = Acute Myocardial Infarction; PNA = pneumonia.

**Table 5:** Impact of process factors on FMC readmission rates.

Factor	Readmission Rate
Follow-Up Appointment in ≤ 7 days	11.8%
Follow-Up Appointment in > 7 days	20.0%
Follow-Up Appointment in ≤ 30 days	14.4%
Follow-Up Appointment in > 30 days	25%
Initial Appointment with PCP	14.9%
Initial Appointment not with PCP	18.8%

**Note:** PCP: primary care provider.

**Table 6:** Impact of an outpatient pharmacist on FMC readmission rates.

Factor	Readmission Rate
Initial Appointment with PharmD	5.0%
Initial Appointment not with PharmD	18.6%
PharmD Appointment ≤ 30 days	6.5%
No PharmD Appointment ≤ 30 days	20.0%
